# Programmable terahertz chip-scale sensing interface with direct digital reconfiguration at sub-wavelength scales

**DOI:** 10.1038/s41467-019-09868-6

**Published:** 2019-06-20

**Authors:** Xue Wu, Huaixi Lu, Kaushik Sengupta

**Affiliations:** Department of Electrical Engineering, Princeton University, Princeton, NJ 08544 USA

**Keywords:** Optical sensors, Terahertz optics, Imaging and sensing, Photonic devices

## Abstract

The ability to sense terahertz waves in a chip-scale technology operable at room temperature has potential for transformative applications in chemical sensing, biomedical imaging, spectroscopy and security. However, terahertz sensors are typically limited in their responsivity to a narrow slice of the incident field properties including frequency, angle of incidence and polarization. Sensor fusions across these field properties can revolutionize THz sensing allowing robustness, versatility and real-time imaging. Here, we present an approach that incorporates frequency, pattern and polarization programmability into a miniaturized chip-scale THz sensor. Through direct programming of a continuous electromagnetic interface at deep subwavelength scales, we demonstrate the ability to program the sensor across the spectrum (0.1–1.0 THz), angle of incidence and polarization simultaneously in a single chip implemented in an industry standard 65-nm CMOS process. The methodology is compatible with other technology substrates that can allow extension of such programmability into other spectral regions.

## Abstract (web summary)

Chip-scale, room-temperature terahertz sensing can potentially enable many applications, but remains restricted in terms of spectrum, angle of incidence and polarization. Here the authors report a methodology for a CMOS-based, programmable THz sensor surface that allows dynamic adaption in all three properties.

## Introduction

The terahertz spectrum, occupying the frequency range between 0.3 and 3 THz, has potential for transformative applications in communication, sensing, spectroscopy, and imaging due to its desirable properties such as non-ionizing photon energy, penetration capability through optically opaque materials, unique spectral signatures for large bio-molecules and chemicals^1–9^. To enable this diverse set of applications, there has been a concerted effort in the research community to miniaturize complex THz systems into chip-scale form that are operable at room temperature. Wedged between the microwave band and the infrared spectrum, this effort has spanned across a large array of substrates ranging from solid-state to photonic devices^10–14^, 2D nano-materials^15,16^, quantum-cascade lasers (QCLs)^17–19^, microbolometers^20,21^, nanowires^22^, metamaterials^23^, and ultrafast photoconductive materials^24,25^. In recent years, even silicon, particularly complementary-metal-oxide-semiconductor (CMOS) based integrated circuits and chips have been demonstrated in the frequency range with power generation capability in the range of 100s of μW^26–29^ and detection capability with sensitivities (noise-equivalent-power) in the sub-100 pW/$\sqrt {{\mathrm{Hz}}}$ region^30–37^. The field-effect-based devices can detect THz waves at frequencies beyond their cutoff frequencies by exploiting nonlinearities in the operation regime through a non quasistatic plasma-wave excitation by the incident THz waves^38^. They also exhibit orders of magnitude much faster response times and higher pixel integration capability compared with microbolometers^32^. This has been a significant advancement, as it not only makes possible THz systems (below 1 THz) compact and battery operable at room temperature, but also exploits the economics of scale of semiconductor fabrication to enable complex THz systems in a cost-effective fashion.

A limiting factor in all of these classes of THz sensors is that they are typically sensitive to a narrow set of the incident field properties, i.e., including a limited spectral range, angle of incidence, and polarization. This has hindered deployment of THz sensors in practical applications where single modality sensing across any field property is not robust enough. Due to the spectrally sensitive nature of scattering and penetration through optically opaque objects in these frequency ranges, sensor fusions exploiting frequency, pattern and polarization diversity, will become increasingly important for high-performance sensing applications. Prior works have demonstrated merging imaging with spectroscopic sensing, exploiting the unique advantages in the THz regime, to clearly differentiate the advantages against other spectral regions^39–41^. We are seeing a similar evolution in the neighboring frequencies, where sensor fusions particularly combining millimeter-wave, infra-red and optical frequencies is becoming critical to enable effective and robust understanding of the environment for autonomous vehicles and systems. In a similar fashion, the ability to extract information across the incident THz field properties can allow a much richer sensing interface^42,43^.

Enabling such programmability in the THz spectrum, particulary in chip-scale form, is very challenging. Limited reconfigurability in THz systems have been demonstrated in mechanically and optically controlled metamaterials^43,44^, micro-electromechanically actuated slits for THz modulation^45^, thermally tunable THz filters^46^ and phase-change material^47^, graphene-based switchable high-impedance surface (HIS)^48,49^, and graphene-based multi-input–multi-output antenna array^50^. The key to realize such a rich sensing interface is to allow simultaneous programmability across all the three incident field properties and across such a wide variation range. In addition, integration in a substrate compatible with semiconductor fabrication processes is also important to allow for low-cost and wide-scale deployments of such sensors. This is particularly challenging, since the THz frequencies can far exceed the cutoff frequencies of such devices making any form of reconfigurability very limited and inefficient.

In this paper, we present our approach toward a universal THz sensing surface which can reconfigure its responsivity to spectrum (0.1–1.0 THz), incidence direction and polarization. We demonstrate the approach in a single chip implemented in an industry standard 65-nm CMOS process with *f*_max_ ≈ 0.25 THz. The metric *f*_max_ designates the frequency where the unilateral gain of the device falls to one^1^. Therefore, this distributed approach, realized with devices operating up to four times their cutoff frequencies, can be translated to other semiconductor platforms for programmability in other spectral ranges. While preliminary results on the sensor response at THz were presented in ref. ^51^, this paper focuses on the design methodology of mapping subwavelength reconfiguration states to the set of incident field parameters including spectrum, angle of incidence and polarization. We also present measurement results in the implemented chip to demonstrate the tri-modal reconfigurability across 0.1–1.0 THz.

## Results

### Direct digital programming of THz surface

The ability to reconfigure against incident field properties opens up new dimensions to information that can be extracted from the sensor interface. Hyperspectral operation can enable simultaneously higher resolution acquisition with higher penetration depth for 3D imaging^39^ due to the spectrally dependent resolution and penetration depth of electromagnetic waves. The ability to electronically scan the beam pattern to various angles of incidence (pattern reconfigurability) can reduce image acquisition time by orders of magnitude circumventing slow mechanical raster scanning. In addition, such pattern diversity can also introduce new orthogonal measurements allowing computational-based techniques for real-time imaging^51–53^. A sensor that can program to orient its reception beam to different angles of incidence is tantamount to phased array operation. While phased arrays have been demonstrated at radio^54^ and optical frequencies^55^, terahertz operation of such arrays have been severely limited due to the unavailability of efficient active components such as amplifiers, phase shifters, and coherent sources which are the critical components of such a system^1^. Further, phased array operation is typically limited to a narrow range of frequencies where the discrete antenna spacings is ~*λ*/2 to avoid aliasing and grating lobes in unwanted directions. At the lower frequency end, spacings with <*λ*/2 spacing reduces aperture area and typically suffers from inter-element mutual coupling that typically limits the array performance. Therefore, achieving simultaneous spectral and pattern reconfigurability has been very challenging, much more so at THz frequencies. In addition, the ability to reconfigure its polarization sensitivity is also important, since polarization rotation typically happens during transmission or reflection-based imaging and in spectroscopy.

Fundamentally, the limitations of a THz sensor to these three THz field properties are dependent on the electromagnetic resonant nature of the interface, its scattering properties and the coupling to the detector, in whichever substrate the latter is realized. First, sensitivity to spectrum arises due to the resonant electromagnetic modes sustained on the antenna surface and the resonant nature of the antenna-detector interface^37^. The latter is designed to ensure optimal power transfer and impedance matching, that is typically guaranteed within a narrow range of frequencies. Broadband detection without frequency selectivity can be achieved in optical domain with photoconductive substrates^7^, but the reception patterns are static and image acquisition requires bulky and complex optical assemblies, including femtosecond lasers. In addition to frequency selectivity, sensitivity to the other field properties namely angle of incidence and polarization also arises out of the antenna structure and the boundary conditions. Since a single-port antenna-detector system is reciprocal, its reception properties can be understood from its transmission properties. When excited, the antenna surface sustains a 2D THz current distribution, that determines all its electromagnetic properties, including its frequency response, beam pattern, and polarization.

Traditional methods of reconfigurability that focus on the system by partitioning into its functional elements such as the antenna, the coupling network and the detectors are limited in their ability to efficiently achieve the desired parameters particularly at THz frequencies. Typically, such architectures focus on one aspect of reconfigurability against the incident field. While prototypical method of partitioning the design space and applying intuition-based approaches allow us to create a step-by-step design methodology, it also limits the space of possible architectures due to the complex interactions of several inter-dependent variables and properties. This is particularly true at THz frequencies where the individual device performance and variability itself is limited.

**Fig. 1:**
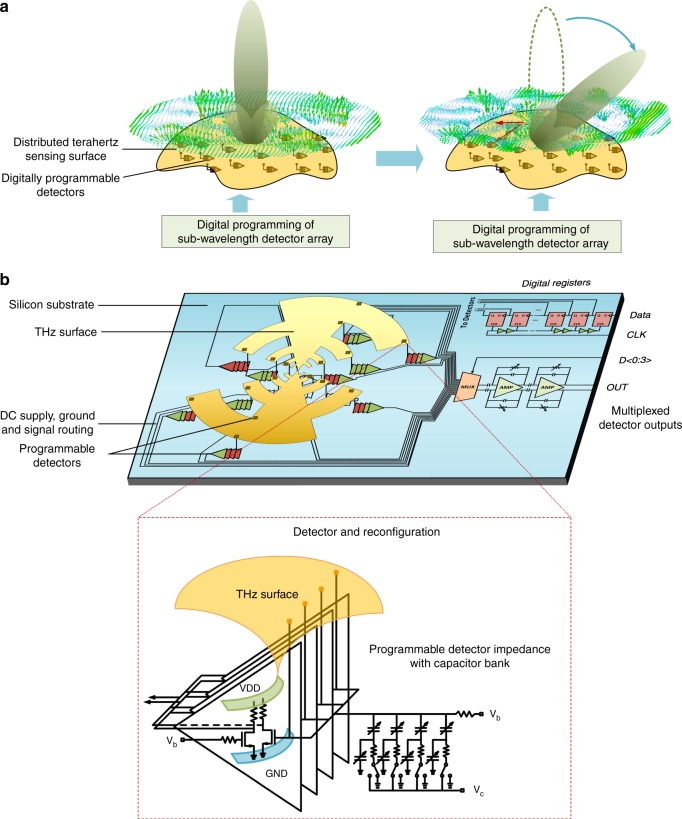
Programmable terahertz chip-scale interface. **a** Concept of an universal terahertz sensing surface that maps a large discerte digital reconfiguration space to incident field properties including spectrum, angle of incidence and polarization. This is acheived by  directly programming the surface currents induced on the interface at subwavelength scales. The incident power is absorbed in a distributed fashion at the sites of reconfiguration. **b** The system overview of the implemented terahertz sensor with a log-periodic tooth interface with 16 detectors distributed over the surface. Each site is reconfigurable with a coded capacitor bank that locally changes the boundary condition to redistribute the surface current distribution. This collectively programs the sensor response to allow for optimal reception against incident spectrum, polarization and angle of incidence

Since the electromagnetic properties of the THz interface is dependent on the THz surface current distribution it supports, the key concept in this work is to directly program the 2D distribution under THz field incidence. This is achieved with active devices placed at subwavelength scales that can simultaneously program and absorb the incident fields at the sites of reconfiguration. Figure 1 shows the concept where we move from a single-antenna-detector interface to a continuous aperture and distributed reconfiguration and absorption. The boundary conditions at each detector site is reconfigurable independently.  This changes the local fields, reprograms the impressed current over the surface and redistributes the power distribution across the detector array. Through the complex interactions of the multiport distributed detector array, the 2D amplitude and phase distribution on the surface is changed. By independently programming the distributed detector array, a large set of THz sensor reception properties can be engineered. The goal of the reconfigurable THz sensor design is to map a subset of these large configuration states against optimal reception across frequency, pattern, and polarization.

Figure 1b illustrates the architecture of the implemented reconfigurable THz surface. Each detector is realized with field-effect-transistors that rectifies the local THz field to produce a signal proportional to the local flux. Each detection site is programmable with a switched capacitor bank and a 4-bit thermometer code. With 16 detectors distributed over the surface, this results in 5^16^ possible reconfigurations of the surface. The locations of the 16 detectors are shown in Fig. 1b. Multiport matching is taken into consideration to enable optimal power absorption into the distributed detector array^33^. The output signals from the 16 detectors are processed on-chip with a chain of integrated analog amplifiers and filters with variable gain. The collective outputs of the detectors that represent the total power absorbed by the sensing surface can be read in a time-multiplexed fashion. The chip is realized in a 65-nm industry standard digital CMOS process and measures 2 mm × 1 mm in dimension. The entire chip dissipates less than 10 mW of DC power.

**Fig. 2:**
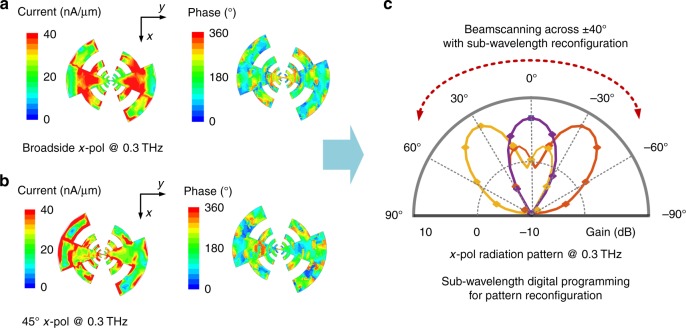
Direct digital programming of the interface against angle of incidence. The figure shows the distribution of amplitude and phase of the impressed surface current under incidence a 0.3 THz at (**a**) broadside and (**b**) at 45° under the optimal detector settings. **c** The simulated beam pattern of the collective absorption across the senor array shows the ability to orient it across various angles of incidence. Functionally, this is similar to a beam-scanning operation of phased arrays. Here, it is realized with a continuous aperture and subwavelength reconfiguration allowing pattern shaping and frequency agility simultaneously

### Mapping of reconfigurable properties to digital states

A log-periodic tooth antenna is used as the sensing surface (see Fig. 1). By moving from a single-port-single-detector system to distributed system, we address three of these reconfigurable properties simultaneously. First, the multiport approach allows us to overcome the bandwidth limitations of the classical 2-port, i.e., single-detector and single-antenna interface. By exploiting mutual interactions of the detectors through the scattering surface, optimal impedances can be synthesized over an order of magnitude higher spectral range. This allows us to increase the Bode-Fano bandwidth limit by a factor of *N*, where *N* is the number of detection sites^56,57^ compared with the classical 2-port case. Intuitively, the antenna surface that accepts the incident field and distributes it across the detector sites simultaneously performs the function of the impedance matching across the distributed detector array. In addition to spectral reconfigurability, since the port reconfiguration and power absorption happens at the same place, it allows the surface current to be programmed to shape the reception beam and polarization. The design goal is to translate these digital states to optimal reception against these incident field properties.

**Fig. 3:**
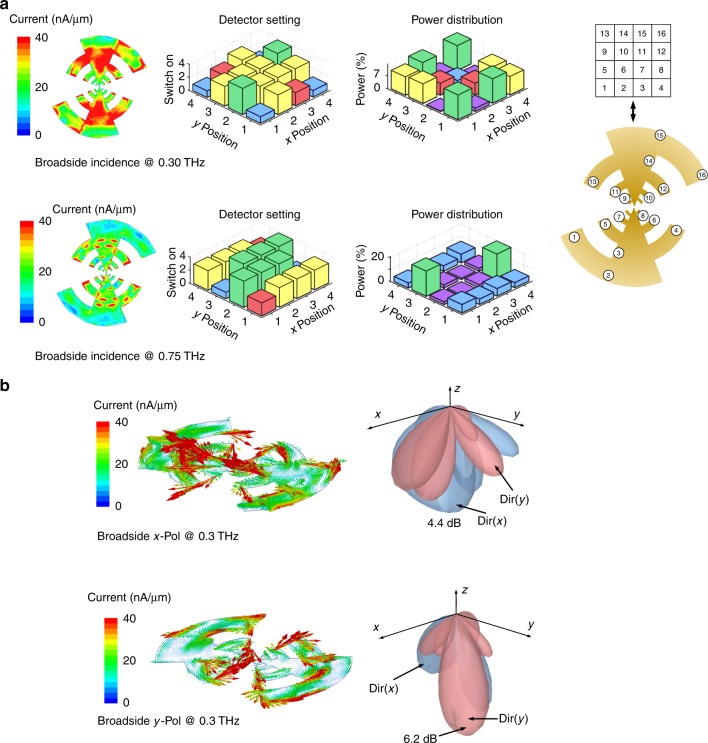
Direct digital programming of the interface against spectrum and polarization. **a** Detector configurations, power distributions and surface current configurations for optimized reception at 0.3 and 0.75 THz at the broadside. The figure also shows the mapping of (*x*, *y*) position to location of the detectors on the antenna. The figure demonstrates that the reprogramming the surface for optimal reception at 0.75 THz can enhance responsivity by 12 times. **b** Simulated polarization responsivity and beam patterns when the surface is actuated against the two polarization. Programming the sensor response can enhance the reception by 16 times through polarization rotation of the surface currents

Figures 2 and 3 illustrate this. We will discuss the methods of optimization of the detector settings later in the paper, but the figure illustrates the effect of subwavelength reconfiguration to THz reception properties. In this setup, the chip is abutted by a silicon lens to suppress the substrate modes. However, to avoid beam narrowing and reducing the reconfiguration range of the patterns in a hyper-hemispherical lens, we use a hemispherical lens with only 0.5 mm of distance kept between the chip and the lens. Figure 2a, b shows the amplitude and phase distribution of the impressed current surface for a linearly polarized incidence at 0.3 THz when the detector settings are optimized for reception for broadside and 45° incidence, respectively. The simulated reception beams in Fig. 2c for these settings (and −45° incidence) demonstrates the ability to electronically shape the reception beam and scan it in space. The sensor responsivity indicates the collective rectified response at the output of the detector array distributed over the surface of the electromagnetic structure. This is first electromagnetically simulated with incident field impinging on the distributed antenna loaded with multiport detector array to extract the voltage (local electric field) at each of the detector ports. Enumerating this allows us to calculate the rectified output response of the detector array with nonlinear circuit simulations. Therefore, the simulation takes into account the entire chain from THz incidence to rectification, including losses in the electromagnetic path, the collective multiport impedance mismatches as well as the detector sensor response at THz frequencies. This is similar to a phased array operation, but realized with a continuous aperture and multiport subwavelength programming. This technique overcomes the sub-sampling nature of classical phased arrays when operated across a wide-frequency range, where the spacing at the highest end can exceed *λ*/2. This can allow simultaneous reconfigurability against frequency and angles of incidence.

Figure 3 illustrates the optimal reception of broadside incidence at frequencies of 0.30 and 0.75 THz showing the detector settings and frequency dependent power absorption across the detector array. Through reprogramming the surface, the sensor achieves up to 12 times enhancement is reception at 0.75 THz as the incident power gets optimally distributed over the detectors. The state reconfiguration can be extended to even polarization as shown in a 16 times increase in reception for the orthogonal polarization with optimized detector settings.

The mapping of detector settings to the space of incident field properties is non-convex. The power absorbed on the surface in such a *N*-port antenna-detector configuration can be derived from the impedance matrices of the passive antenna (**Z**_**Ant**_ ∈ **R**^**N**×**N**^), detectors (**Z**_**L**_ ∈ **R**^**N**×N^), and the open circuit voltages (**V** ∈ **R**^**N**×**1**^) excited at the antenna interface under the particular frequency, polarization, and angle of incidence. As a result, the power captured by the antenna, (*P*_rec_) can be expressed as a function of **Z**_**L**_ as (Supplementary Notes 1–3)

1 $$P_{{\mathrm{rec}}} = \frac{1}{2}{\mathrm{Re}}({\mathrm{V}}^{\bf{H}} ({\mathbf{Z}}_{{\mathbf{Ant}}}^{{\hskip 10pt}\mathbf{H}} + {\mathbf{Z}}_{\mathbf{L}}^{\mathbf{{\hskip 3pt} H}})^{ - 1}{\mathbf{Z}}_{\mathbf{L}}^{\mathbf{{\hskip 3pt} H}}({\mathbf{Z}}_{{\mathbf{Ant}}} + {\mathbf{Z}}_{\mathbf{L}})^{ - 1}{\mathrm{V}})$$

Therefore, the responsivity of this multiport detector system is simulated combining both electromagnetic and nonlinear circuit simulations. The structure is simulated in a 3D electromagnetic simulation tool with incident field to calculate its S-parameter and the open circuit voltages. The absorbed power into the individual ports is then calculated by loading the ports with the impedances of the detectors and the capacitor banks. Once the THz power absorbed at each port is known, the output response of each detector can also be enumerated from the responsivity of each detector through nonlinear circuit simulations (Supplementary Note 1).

Therefore, optimal programming of the THz surface amounts to searching for a diagonal matrix, **Z**_**L**_ in a discrete space to maximize *P*_rec_. Prior works on electromagnetic optimization in such non-convex and discrete space have utilized genetic algorithm^58^, particle swarm optimization^59^, and alternating direction method of multipliers (ADMM)^60^. Here, we combine gradient decent optimization method with a random search algorithm in the 16-dimensional space (formed by the 16 reconfigurable detectors). The discreteness of the space is generated from the digital states of the detectors. Here, optimization is allowed to start with multiple random initial conditions and gradient descent is utilized to obtain the locally optimized solution. In each such iteration, we maximize the total sensor responsivity that is collective response of all the sensors. The process is repeated with a random initial sensor settings to randomize the effect of initial settings.

**Fig. 4:**
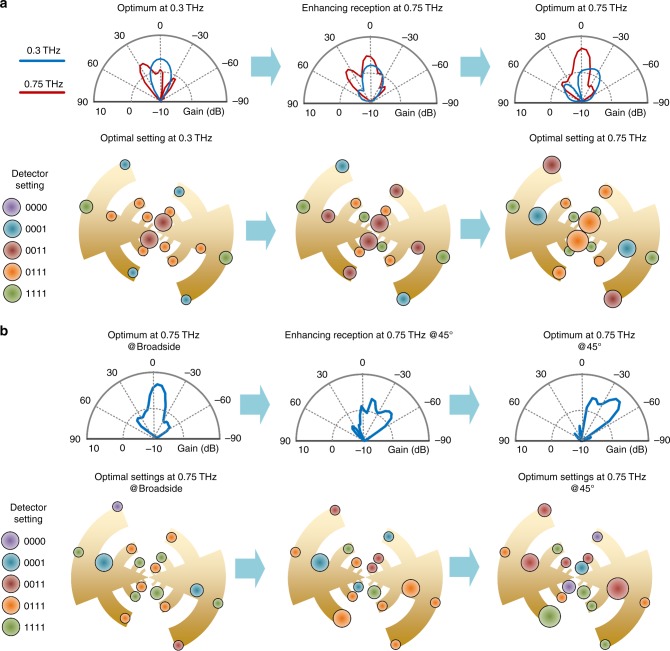
Mapping the digital electromagnetic states to the incident THz sensor response. **a** Transformation of the beam patterns as the detector settings are reprogrammed across the 16-dimensional space from optimal reception from broadside at 0.3–0.75 THz. The beam patterns at both the frequencies can be seen to be transformed at the sensor is reprogrammed to shape its 0.75 THz pattern in the broadside. **b** Similar transformation of the reception beam at 0.75 THz as the surface is reconfigured from optimal reception at broadside to 45° incidence at 0.75 THz

To further understand the transformation of the THz sensor properties as the surface is reconfigured, Fig. 4 illustrates the gradual change of the THz reception pattern from an initial setting to the final optimal one. As shown in Fig. 4a, when the detector setting is optimized for receiving linearly polarized incidence at 0.3 THz, the reception beam for 0.3 THz is expectedly broadside. For the same setting, the sensor responsivity toward a broadside incidence at 0.75 THz is, however, poor as shown in the corresponding pattern. The switch settings are color coded and the sizes of the markers qualitatively represent the magnitude of local absorption of the incident power. To demonstrate the transformation of the reception properties as the detectors are optimized for reception at 0.75 THz (from the earlier setting corresponding to 0.3 THz), we pick an intermediate setting and the corresponding pattern and detector settings are shown. As the surface is programmed, the power distribution and the beam shape at both the frequencies change. The shaping of the beam continues through out the process till it assumes a broadside pattern of 0.75 THz. In this case, the reception of the 0.3 THz beam gradually becomes poor, though that does not necessarily have to occur in a single-objective optimization. However, such surface reconfiguration can also be done to assume a frequency selective character that aims to enhance reception at one frequency and minimize that at another frequency. Such a tunable frequency reception across 0.1–1 THz can allow image extraction at discrete frequency slices with high signal-to-noise ratio (SNR). In comparison, in pulse-based broadband imaging, the incident signal is spread over the spectrum and can lead to lower SNR. The shaping of the reception patterns to tilt the beam to a different angle is shown in Fig. 4b. In a manner similar to the previous case, the local field manipulation reprograms the surface current and the spatial distribution of the incident power, and tilts the reception pattern to allow sensitivity alignment to a different angle, similar to a phased array operation.

### Design methodology

**Fig. 5:**
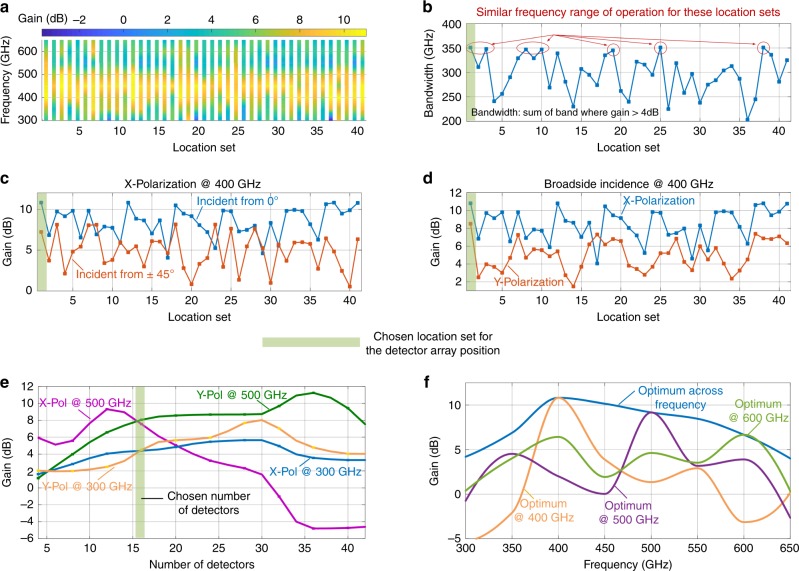
THz response reconfigurability and its trade-off with the location and number of reconfiguration sites. **a** Simulated optimized gain with varying locations of the 16 detectors and its effect on peak responsivity and frequency range of operation. **b** Simulated variations of bandwidth with location sets of the 16 detectors. The figure demonstrates that more than one solution set can exist to achieve a similar frequency range of operation. **c**, **d** Simulated optimized gain with varying locations of the 16 detectors for different angles of incidence and polarization at 0.4 THz. **e** Simulated gain variations with the number of detectors for various incidence fields. **f** For the final design, the figure shows the instantaneous frequency response as the sensor is optimized for various peak frequencies. The optimal reconfigurable sensor response of the chip follows the peak of these individual frequency response curves

As shown through the simulated results, the distributed approach allows simultaneous control of the incident field properties, but it also makes the design space inter-connected where combination of intuitive design and algorithmic-based approaches have to be relied upon. This is particularly true since the choice of the number of detectors and locations collectively influences the sensor response. We choose the surface as a log-periodic tooth antenna to allow the spectrally distributed resonance over the surface. Of course, this changes when interfaced with the distributed detector array. We follow a heuristic based design methodology to choose the number and location of the detector array. This is illustrated in Fig. 5.

The effect of location of the detector array on the frequency reconfigurability is shown in Fig. 5a. Here, the gain of the aperture (*G*_Ant_) is defined similar to a single-port antenna as $P_{{\mathrm{rec}}} = G_{{\mathrm{Ant}}}P_{{\mathrm{flux}}}\frac{{\lambda ^2}}{{4{\mathrm{\pi }}}}$, where *P*_rec_ is the power absorbed by the detectors and *P*_flux_ is the incident flux at wavelength *λ*. We discretize the surface of the radiator into 84 locations at deep subwavelength spacings. We then analyze the effect of the THz sensor response across frequency, angle of incidence and polarization as we choose a subset of 16 locations from them. The locations of the detectors is chosen with a centro-symmetric distribution, that allows simple rotation of the reception beam by 180° across the broadside axis by simply rotating the detector settings. The effect of the location distribution is represented in Fig. 5a, b as the effective gain of the aperture against frequency for a sample set of 40 locations. For each location set, the performance against frequency is analyzed through optimization of the detector settings. While the effect of the locations on the frequency response is clearly seen, there are multiple location positions that allow similar range of frequency reconfigurability as expected. When analyzed against angle of incidence (0°, ±45°) and polarization (*X* and *Y*), the location sets show divergent performances. We choose a location set that allow collectively higher gain (and therefore responsivity) across all the three properties, as show in Fig. 5a–d.

The effect of number of detectors is investigated by increasing the number of locations occupied by the detectors up to 84, and analyzing their reception properties against the incident field. A sampled variation for two frequencies at 300 and 500 GHz and for two polarizations is shown in Fig. 5e. Evidently, lower number of detectors do not show enough reconfigurability across both frequency and polarization. Increasing the number of detectors will load the surface to a large extent effectively reflecting off the incident wave. We choose the number of detectors that allow overall higher response against all the three incident field properties. Once the detector configurations are optimized for a given incident field, Fig. 5f shows the sensor response as the frequency is varied. The optimally reconfigured sensor response traces the peak of these individual curves. In this design, there is no explicit matching network between the multiport scatterer and multiport detector array, and bandwidth is compared with a single-port antenna with second and fourth order matching networks and the reconfigurable range for the multiport structure far exceeds that of the single-port design (Supplementary Note 3).

There is no provable guarantee on the global optimality of the solution, like in most such heuristic and optimization-based design methodologies. However, in many such methods, the approach allows us to break off from the traditional intuitive-based design processes to incorporate reasonably high performance across a wide range of reconfiguration, difficult to achieve otherwise.

### Measurement

**Fig. 6:**
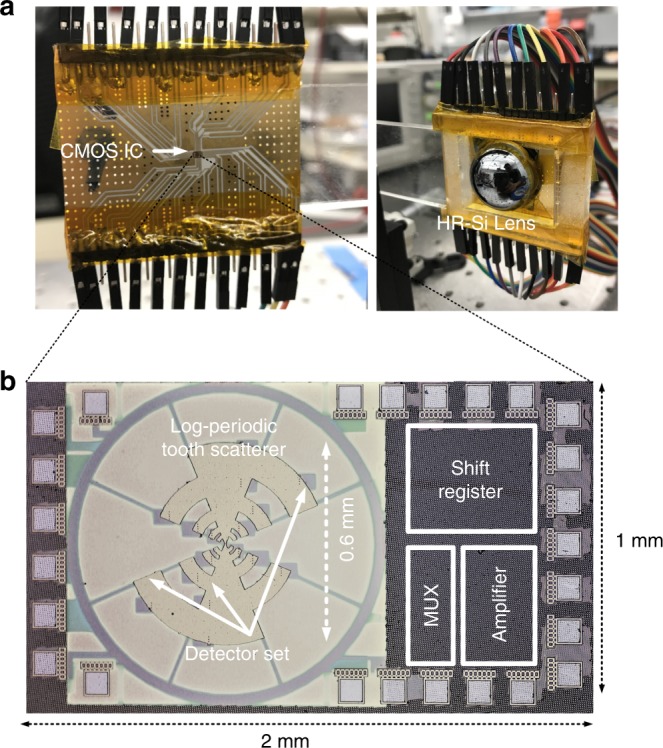
The chip photo and the packaged system. **a** The chip is packaged on a printed circuit board supported by a silicon wafer at the back. A high-resistivity silicon lens abuts the back of the silicon wafer to enable reception of the THz waves from the backside. **b** The chip is implemented in an industry standard 65-nm CMOS process and measures 2 mm × 1 mm in dimension

The chip is implemented in a 65-nm CMOS process. All measurements are carried out at room temperature. During measurement, the chip is mounted vertically on a Rogers printed circuit board with a high-resistivity silicon wafer at the backside. A high-resistivity silicon lens abuts the wafer at the backside of the substrate to suppress substrate modes and the signal is irradiated from the backside (Fig. 6a). In a manner similar to the simulations, we use a hemispherical lens with a 0.5 mm distance between the chip and the lens to avoid the sharp narrowing of the beam in a hyper-hemispherical lens setup. The chip occupies an area of 2 mm × 1 mm as illustrated in Fig. 6b. More  details can be found   in Supplementary Note 4.

**Fig. 7:**
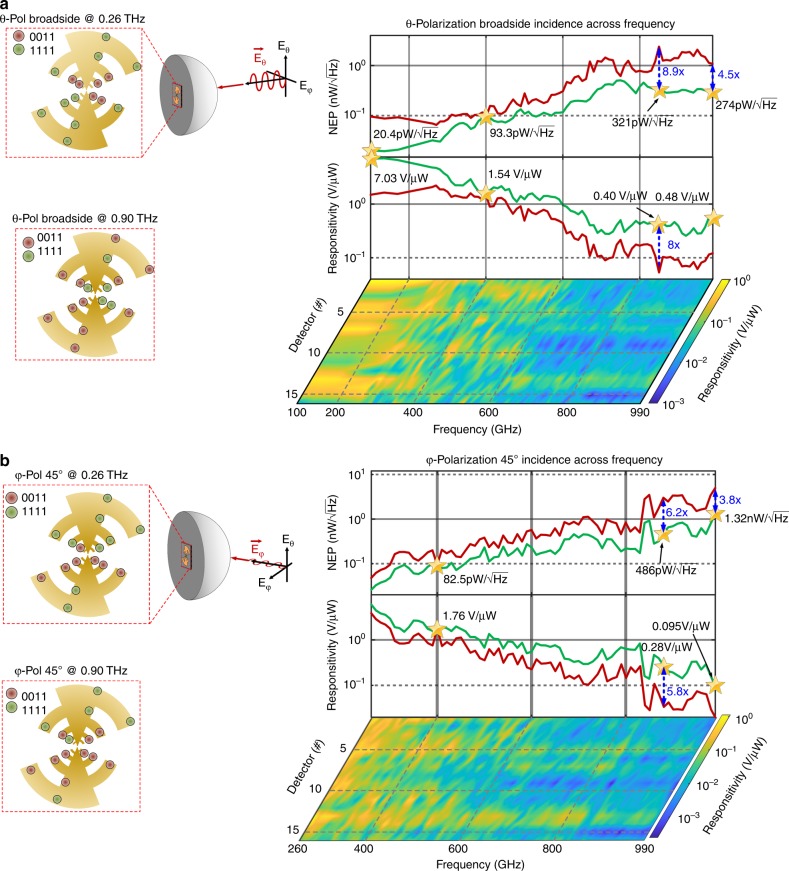
Measurement results for optimized responsivity and corresponding NEP across 0.1–1.0 THz for two incidence angles at **a** 0° and **b** 45°, and two polarizations (*E*_*θ*_ and *E*_*φ*_). The figure shows 8.9 and 4.5 times reduction in NEP at 0.86 THz and at 0.99 THz for broadside incidence and 6.2 times reduction in NEP for 45° incidence at 0.87 THz. The green line represents the sensor performance as it is optimized for each incident frequency. The red line represents the performance across frequency for a fixed optimal configuration at 0.4 THz. The 2D color plots represent the contribution of power incident absorbed across the detector array across the optimized states between 0.1-1.0 THz. The figure also shows four examples of optimized states for the incident field impinging at two angles of incidence at 0.26 THz and 0.9 THz

**Fig. 8:**
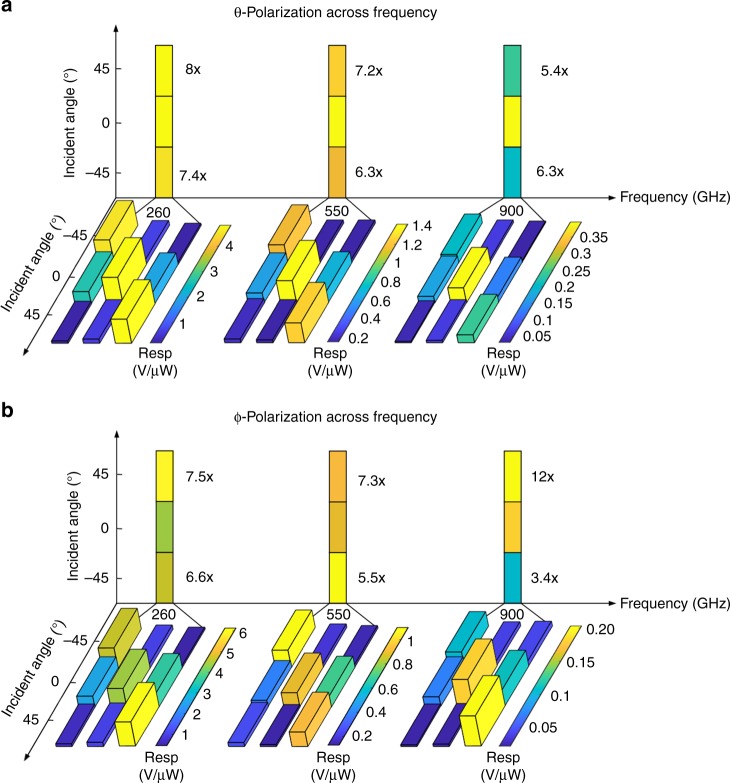
Measured sensor beam programming with subwavelength reconfiguration. This is measured with varying angles of incidence for two polarizations across 0.1–1.0 THz. **a** Measured optimized performance as the incidence angle is varied for two polarizations across 0.10–1.0 THz. For both *E*_*θ*_ and *E*_*φ*_, the sensitivity can be enhanced significantly by 5–12 times across 0.10–1.0 THz. The horizontal plane shows the instantaneous beam configuration when optimized for reception across the various angles of incidence (45°, 0° and −45°). As can be seen,  the reprogramming of the detector states redistributes the surface current to effectively tilt the beam toward the desired angles of incidence. The vertical plane shows the overall responsivitiy enhancement as a result of the reconfiguration when compared against the optimal normal incident state. **b** This is also shown in the significant enhancement with reprogramming as the polarization is switched across *E*_*θ*_ to *E*_*φ*_ across the range

The performance of the chip across the incidence field properties across 0.1–1.0 THz is shown in Figs. 7 and 8. During measurement, optimization to reconfigure against the incident THz field properties is carried out in the following fashion. At the beginning, the sensor surface is actuated with a random detector array setting through a 64-bit shift register. As the optimization process is executed, sensor outputs are automatically acquired for each setting after they have stabilized. We apply gradient descent optimization on the acquired data on an external computer and the sensor is reprogrammed. The process is repeated with a random initial sensor settings to scramble the effects of initial settings. The entire optimization process is fully automated. In order to reduce the space of the optimization in measurement, we choose three states per detector (instead of five) that corresponds to a total of more than 43 million possible configurations.

The large reconfiguration space is key toward simultaneous optimization against spectrum, angle of incidence and polarization. The measured optimized responsivity and noise-equivalent-power (NEP) across 0.1–1.0 THz for two incidence angles (0° and 45°) and two polarization (*E*_*θ*_ and *E*_*φ*_) are demonstrated in Fig. 7. The figure compares the optimized performance across the frequency range against a static configuration for broadside incidence at 0.4 THz. The green line represents the overall sensor performance as the detector states are optimized for each incident frequency. The red line represents the sensor performance across frequency for a fixed optimal configuration at 0.4 THz. As can be seen, active reprogramming of the sensing surface allows significant enhancement of sensitivity, including 8.9 and 4.5 times reduction in NEP at 0.86 and 1.0 THz for broadside incidence with *θ* polarized waves. We can see a similar enhancement in responsivity and reduction in NEP for 45° incidence with *ϕ* polarized waves, including a 6.2 times reduction in NEP at 0.87 THz. The optimized sensor achieves NEP of $20.4\,{\mathrm{pW}}/\sqrt {{\mathrm{Hz}}} - 274$ ${\mathrm{pW}}/\sqrt {{\mathrm{Hz}}}$ across 0.10–1.0 THz at broadside incidence with *θ* polarized waves and $25.9\,{\mathrm{pW}}/\sqrt {{\mathrm{Hz}}} - 1.32\,{\mathrm{nW/}}\sqrt {{\mathrm{Hz}}}$ at 45° angle of incidence with *ϕ* polarized waves across 0.26–1.0 THz. The figure also shows the spectrally dependent distribution of the absorbed power across the detector array as the incident frequency is varied. The spectrally dependent nature of the distribution of the incident power is evident. The figure also shows the optimized detector settings for the two cases at 0.26 and 0.90 THz.

To demonstrate the ability to tilt the beam and reprogram the polarization sensitivity with subwavelength programming, we test the chip by varying the incidence angle for two polarizations, *E*_*θ*_ and *E*_*φ*_ across 0.10–1.0 THz as shown in Fig. 8. Figure 8a shows the sensor responsivity across angles of incidence as the system is reconfigured successively for incidence angles varying from −45° to 45°. Here, we show the responsivity across incidence angles as the beam pattern is reconfigured to tilt from −45° to 45°. We also show the overall reconfigured response across angles that allows the sensor to achieve a high response across wide angles of incidence, while being directive for a given configuration. As shown in the Fig. 8a, at ±45° incidence, the responsivity can be enhanced by 5–12 times nearly reaching similar responsivity as achieved with broadside incidence. The programming of the surface effectively tilts the reception beam towards the angle of incidence allowing electronic scanning across 0.1–1.0 THz for a wide field of view without sacrificing directivity. It is possible to utilize the establishment of such multiple reception beams across the spectrum to create orthogonal measurements for computational-based real-time imaging, though we have not implemented this in this work^53^.

Such reconfiguration can be achieved if we vary the polarization keeping the spectrum and angle of incidence constant. Figure 8b shows that the significant enhancement across the frequency range can be observed with reprogramming as we switch the polarization from *E*_*θ*_ to *E*_*φ*_, reaching up to 12 times as the subwavelength reconfiguration re-orients the beam. The achievable performance obtained across spectrum, angle of incidence and polarization, are comparable to a collection of state-of-art sensors, each custom designed within a specified range of these parameters^30–32,36,37^. The experiments demonstrate the ability of such multiport electromagnetic structures with direct programming of the sensing interface to allow for dynamic optimization against incident field properties. Combining these ranges of reconfigurability with sensor fusions can allow for low-cost, robust, versatile, and compact THz sensing and imaging technology.

## Discussion

The ability to allow a single chip-scale THz sensor to reconfigure simultaneously against the incident field properties can significantly advance THz sensing and imaging. The sensing surface, described here, allows reconfiguration for reception across 0.1–1 THz with beam-scanning ability across ±45° for both polarizations. Being able to sense THz waves with high sensitivity across 10:1 frequency ratio can enable hyperspectral imaging with both high lateral and depth resolution. Sensor fusions across multiple orthogonal domains (including frequency, beams, and polarization) can allow for superior image registration, and is steadily growing in importance in other spectral domains. This degree of reconfigurability is critical for practical THz sensing systems, where a single modality interface typically does not suffice. By moving from a single-antenna and single-detector interface to a multiport continuous aperture, it is possible to overcome the grating lobe issues allowing frequency and pattern diversity at the same time. This can allow fast image acquisition, hyperspectral and multi-angle imaging, and computational techniques in the THz regime for real-time imaging.

The challenge to engineer such programmability is exacerbated by the fact that chip-scale devices are limited in their cutoff frequencies (*f*_max_). In the implemented chip, the optimal device *f*_max_ is ~0.25 THz, implying that the devices operate close to four times their cutoff frequencies, when operating near 1 THz. Therefore, the presented method that allows such reconfigurable properties at frequencies beyond *f*_max_ can be applied to III–V compound semiconductor-base devices to extend operation into higher THz frequencies (~3–5 THz).

The approach of mapping electromagnetic and sensor properties from a large reconfiguration set also opens up a new design space. By merging multiple functionalities within a single electromagnetic structure (such as, impedance matching and radiating surface in this work), new sensor architectures can emerge. Optimization methods in passive device design, albeit in the photonic domain, has been shown to have yield competitive performance against classical design approaches, but has the potential to open up a new class of optical devices^60^. The computational and distributed subwavelength sensing approach also opens up previously unexplored questions on optimal antenna shapes and detector co-design methodologies in such massively multiport structures, and also new techniques and systems in the optical domain^61–63^. Unlike a static antenna whose shape and boundary condition determine its electromagnetic properties, here, both the structure and the detectors, including their sizing and locations determine the overall responsivity. While we choose a log-periodic structure as the THz sensing surface, its use for spectral reconfigurability is quite unlike its more traditional use as a wideband interface with a single center port excitation. In fact, in this work, no signal is drawn from the center port at all (see Fig. 1). It can be noted that while in this work we investigate the position, location, and configuration of the detector set for the desired set of reconfigurable parameters based on the choice of the log-periodic tooth surface, an interesting direction for future exploration is the space of co-design of the geometry of the surface and sensory interface.

Given the importance of reconfigurability and sensor fusion for robust THz imaging and sensing, we expect the space of programmable designs can lead to future THz sensors with added capabilities. While evidently, this is not as frequency selective as a coherent system, it does overcome the frequency range limited by the tunable nature of such sources in addition to the reconfigurability against incidence angle and polarization. However, this trade-off space is worth investigating for future work to explore further the design parameters and their cross-dependence beyond the ones we have addressed in this work.

The implementation of the chip in a commercial CMOS foundry is a significant advancement in demonstrating such programmable sensors operation up to 1 THz. Their operation at room temperature with extremely low power is also important for future applications where power, compactness, and programmability are critical considerations. Eliminating complex optical instrumentation and integration of such programmable functions in a chip-scale form is expected to have significant impact in both technology and application development in the THz spectrum.

## Methods

### Integrated chip implementation and measurement details

Details of the integrated circuit design and the component designs are provided in Supplementary Notes 1, 2 and 3. The log-periodic tooth antenna is realized on a 1.4-μm thick aluminum layer inside the CMOS chip utilizing the embedded metal interconnect layers. The detectors are realized using 65-nm CMOS transistors and the details are shown in Fig. 1 and Supplementary Fig. 1. The biasing voltage and resistors are optimized for minimizing NEP across 0.1–1 THz (Supplementary Figs. 2 and 3). The digitally controllable impedance are realized with capacitor banks with multiple digitally controlled NFETs in parallel as shown in Supplementary Fig. 4. The control signal of the capacitor bank is a 4-bit thermometer code provided by the 64-bit shift register. The chopped rectified THz signals are processed on-chip with integrated amplifiers (Supplementary Fig. 5). They are realized with integrated transistors as shown in Supplementary Fig. 6. The gain is electronically controllable with a variation of 35 dB (Supplementary Fig. 7). The multiport antenna structure, its interface with detectors and calculations of power absorption under THz field incidence is explained in the Supplementary (Supplementary Figs 8 and 9). The measurement setup is shown in Supplementary Fig. 10. The signals are generated with frequency multiplier bank across 0.1–1 THz and across various angles of incidence with a motor-controlled stage. The signal generator is locked to an external 10 MHz signal that provides the reference for lock-in amplifier. The chip is programmed with a Nexs 4 FPGA and the lock-in data is read in a computer through the GPIB ports. The entre system is automated for fully autonomous optimization against the incidence field properties.

## Supplementary information

Supplementary Information

## Data Availability

The data that support the findings of this study are available from the corresponding author upon reasonable request.

## References

[1] Sengupta K, Nagatsuma T, Mittleman D (2018). Terahertz integrated electronic and hybrid electronic-photonic systems. Nat. Electron..

[2] Duling I, Zimdars D (2009). Terahertz imaging: revealing hidden defects. Nat. Photonics.

[3] Song HJ, Nagatsuma T (2011). Present and future of terahertz communications. IEEE Trans. THz Sci. Technol..

[4] Nagatsuma T, Ducournau G, Renuad C (2016). Advances in terahertz communications accelerated by photonics. Nat. Photonics.

[5] Woolard DL (2005). Terahertz frequency sensing and imaging: a time of reckoning future applications. Proc. IEEE.

[6] Pickwell E, Wallace VP (2006). Biomedical applications of terahertz technology. J. Phys. D. Appl. Phys..

[7] Liu HB (2007). Terahertz spectroscopy and imaging for defense and security applications. Proc. IEEE.

[8] Xie L (2015). Extraordinary sensitivity enhancement by metasurfaces in terahertz detection of antibiotics. Nat. Sci. Rep..

[9] Cooper KB (2011). THz imaging radar for standooff personnel screening. IEEE Trans. THz Sci. Technol..

[10] Stake J, Malko A, Bryllert T, Vukusic J (2017). Status and prospects of high-power heterostructure barrier varactor frequency multipliers. Proc. IEEE.

[11] Urteaga M, Griffith Z, Seo M, Hacker J, Rodwell MJW (2017). InP HBT technologies for THz integrated circuits. Proc. IEEE.

[12] Chevalier P (2017). Si/SiGe:C and InP/GaAsSb heterojunction bipolar transistors for THz applications. Proc. IEEE.

[13] Voinigescu SP (2017). Silicon millimeter-wave, terahertz, and high-speed fiber-optic device and benchmark circuit scaling through the 2030 ITRS horizon. Proc. IEEE.

[14] Schröter M (2017). SiGe HBT technology: future trends and TCAD-based roadmap. Proc. IEEE.

[15] Ju L (2011). Graphene plasmonics for tunable terahertz metamaterials. Nat. NanoTech..

[16] Tassin P (2013). Graphene for terahertz applications. Science.

[17] Vijayraghavan K (2013). Broadly tunable terahertz generation in mid-infrared quantum cascade lasers. Nat. Comm..

[18] Sirtori C, Barieri S, Colombelli R (2013). Wave engineering with THz quantum cascade lasers. Nat. Photon..

[19] Wanke MC (2010). Monolithically integrated solid-state terahertz transceivers. Nat. Photon..

[20] Hammar A (2011). Terahertz direct detection in YBa_2_Cu_3_O_7_ microbolometers. IEEE Trans. THz Sci. Technol..

[21] Liu L (2009). Development of integrated terahertz broadband detectors utilizing superconducting hot-electron bolometers. IEEE Trans. Appl. Supercond..

[22] Peng K (2014). Single nanowire photoconductive terahertz detectors. Nano. Lett..

[23] Grady N. K., Heyes J. E., Chowdhury D. R., Zeng Y., Reiten M. T., Azad A. K., Taylor A. J., Dalvit D. A. R., Chen H.-T. (2013). Terahertz Metamaterials for Linear Polarization Conversion and Anomalous Refraction. Science.

[24] Tanoto H (2012). Greatly enhanced continuous-wave terahertz emission by nano-electrodes in a photoconductive photomixer. Nat. Photon..

[25] Heshmat B (2012). Nanoplasmonic terahertz photoconductive switch on GaAs. Nano. Lett..

[26] Huang, D. et al. 324 GHz CMOS frequency generator using linear superposition technique. *IEEE Int. Solid-State Circuits Conf. Dig. Tech. Papers (ISSCC)*, 476–477 (2008).

[27] Hu Z, Kaynak M, Han R (2018). High-power radiation at 1 THz in silicon: a fully scalable array using a multi-functional radiating mesh structure. IEEE J. Solid-State Circuits.

[28] Sengupta K, Hajimiri A (2012). 0.28 THz power-generation and beam-steering array in CMOS based on distributed active radiators. IEEE J. Solid-State Circuits.

[29] Momeni O, Afshari E (2011). High power terahertz and millimeter-wave oscillator design: a systematic approach. IEEE J. Solid-State Circuits.

[30] Liu ZY, Liu LY, Yang J, Wu NJ (2017). A CMOS fully integrated 860-GHz terahertz sensor. IEEE Trans. THz Sc. Techn..

[31] Han R (2013). Active terahertz imaging using Schottky diodes in CMOS: array and 860-GHz pixel. IEEE J. Solid-State Circuits.

[32] Hadi R (2012). A 1 k-pixel video camera for 0.7–1.1 terahertz imaging applications in 65-nm CMOS. IEEE J. Solid-State Circuits.

[33] Wu. X, Sengupta K (2016). On-chip THz spectroscope exploiting electromagnetic scattering with multi-port antenna. IEEE J. Solid-State Circuits.

[34] Wu X, Sengupta K (2018). Single-chip source-free terahertz spectroscope across 0.04–0.99 THz: combining sub-wavelength near-field sensing and regression analysis. Opt. Exp..

[35] Wu, X. & Sengupta, K. Wide-band THz spectroscope in silicon THz combining sub-wavelength near-field sensing and robust regression analysis. *Intl. Microw. Symp*. 1468–1471 (2018). 10.1364/OE.26.007163 29609403

[36] Schuster F (2011). Broadband terahertz imaging with highly sensitive silicon CMOS detectors. Opt. Express.

[37] Sengupta K, Seo DJ, Yang L, Hajimiri A (2015). Silicon integrated 280 GHz imaging chipset with 4 × 4 SiGe receiver array and CMOS source. IEEE Trans. THz Sc. Techn..

[38] Dyakonov M, Shur M (1993). Shallow water analogy for a ballistic field effect transistor: New mechanism of plasma wave generation by DC current. Phys. Rev. Lett..

[39] Xu Z, Lam EY (2010). Image reconstruction using spectroscopic and hyperspectral information for compressive terahertz imaging. J. Opt. Soc. Am. A..

[40] Hong L, Sengupta K (2017). Fully Integrated optical spectrometer between 500 nm–830 nm in CMOS. IEEE Trans. Biomed. Circuits Syst. (Spec. ISSCC issue).

[41] Hong, L. & Sengupta, K. Fully integrated optical spectrometer with 500-to-830 nm range in 65 nm CMOS. *IEEE Intl. Conf. Solid-State Circuits Conf*. 462–463 (2017).

[42] Sensale-Rodriguez B, Yan R, Liu L, Jena D, Xing HG (2013). Graphene for reconfigurable terahertz optoelectronics. Proc. IEEE.

[43] Tao, H. et al. Reconfigurable terahertz metamaterials. *Phys. Rev. Lett*., **103**, 147401 (2009). 10.1103/PhysRevLett.103.147401 19905602

[44] Chen HT (2008). Experimental demonstration of frequency-agile terahertz metamaterials. Nat. Photon..

[45] Berry CW, Moore J, Jarrahi M (2011). Design of reconfigurable metallic slits for terahertz beam modulation. Opt. Exp..

[46] Liu C, Ye J, Zhang Y (2010). Thermally tunable THz filter made of semiconductors. Opt. Comm..

[47] Sanphuang V, Ghalichechian N, Nahar NK, Volakis JL (2016). Reconfigurable THz filters using phase-change material and integrated heater. IEEE Trans. THz. Sci. Technol..

[48] Huang Y, Wu LS, Tang M, Mao J (2012). Design of a beam reconfigurable THz antenna with graphene-based switchable high-impedance surface. IEEE Trans. Nano..

[49] Wang XC, Zhao WS, Hu J, Yin WY (2015). Reconfigurable terahertz leaky-wave antenna using graphene-based high-impedance surface. IEEE Trans. Nano..

[50] Xu Z, Dong X, Bornemann J (2014). Design of a reconfigurable MIMO system for THz communications based on graphene antennas. IEEE Trans. THz Sci. Technol..

[51] Wu, X. & Sengupta, K. A programmable active THz electromagnetic surface on-chip for multi-functional imaging. *Intl. Microw. Symp*. 1464–1467 (2018).

[52] Watts CM (2014). Terahertz compressive imaging with metamaterial spatial light modulators. Nat. Photon..

[53] Hunt J (2013). Metamaterial apertures for computational imaging. Sciece.

[54] Sadhu B (2017). A 28-GHz 32-element TRX phased-array IC with concurrent dual-polarized operation and orthogonal phase and gain control for 5G communications. IEEE J. Solid-State Circuits.

[55] Sun J (2013). Large-scale nanophotonic phased array. Nature.

[56] Nie D, Hochwald BM (2017). Improved broadband matching bound. IEEE Trans. Antenna Propag..

[57] Nie D, Hochwald BM (2015). Broadband matching bounds for coupled loads. IEEE Trans. Circuits Syst..

[58] Rahmat-Samii Y, Michielssen E (1999). Electromagnetic Optimization by Genetic Algorithms..

[59] Robinson J, Rahmat-Samii Y (2004). Particle swarm optimization in electromagnetics. IEEE Trans. Antenna Propag..

[60] Piggott AY (2015). Inverse design and demonstration of a compact and broadband on-chip wavelength demultiplexer. Nat. Photon..

[61] Hong L, Li H, Yang H, Sengupta K (2017). Fully integrated fluorescence biosensors on-chip employing multi-functional nanoplasmonic optical structures in CMOS. IEEE J. Solid-State Circuits.

[62] Hong L, Li H, Yang H, Sengupta K (2018). Nano-plasmonics and electronics co-integration in CMOS enabling a pill-sized multiplexed fluorescence microarray system. Biomed. Opt. Exp..

[63] Hong L, Li H, Yang H, Sengupta K (2018). Integrated copper-based sub-wavelength angle insensitive nano-plasmonic filters for ultra-miniaturized fluorescence microarray in a 65-nm digital CMOS process. Acs. Photonics.

