# Supplementary material for: Programmable terahertz chip-scale sensing interface with direct digital reconfiguration at sub-wavelength scales
Source: Nat Commun. 2019 Jun 20;10:2722. doi: 10.1038/s41467-019-09868-6 (PMC6586644; doi:10.1038/s41467-019-09868-6)
Supplement: Supplementary file 1 — Supplementary Information [file 41467_2019_9868_MOESM1_ESM.pdf]

Supplementary Information: Programmable terahertz chip-scale sensing interface with direct digital reconfiguration at sub-wavelength scales

Xue Wu, Huaixi Lu and Kaushik Sengupta, Department of Electrical Engineering, Princeton University, Princeton, NJ 08544.

1 Supplementary Note 1: Digitally Controlled Reconfigurable Detector

The overall concept of the reconfigurable THz sensor is demonstrated in Supplementary Fig. 1. The total incident power on the surface is the collective power absorbed into the detector array ($P_{\text{rec}} = \sum_i P_i$). A log-periodic tooth antenna with a radius of 0.3 mm is selected to be the radiating surface. 16 THz detectors with digitally controlled impedances are distributed underneath the THz sensing surface, and the states are programmable by a 64-bit shift register. A baseband amplifier chain processes the detector signals before digitization. Supplementary Fig. 2 shows the schematic of the programmable detector which consists of 4 sub-detectors in parallel with a switchable capacitor bank for impedance modulation.

The detector consists of two 3μm/65nm NFETs biased in cut-off region to convert RF power into DC signal efficiently. As shown in Supplementary Fig. 2, the design is optimized to provide the highest sensitivity across the frequency range (0.10-1.0 THz). Supplementary Fig. 3 demonstrates the simulated responsivity and NEP across the frequency range for a single detector. The responsivity varies from 18 mV/μW to 0.8 mV/μW across 0.1-1.0 THz. The corresponding NEP

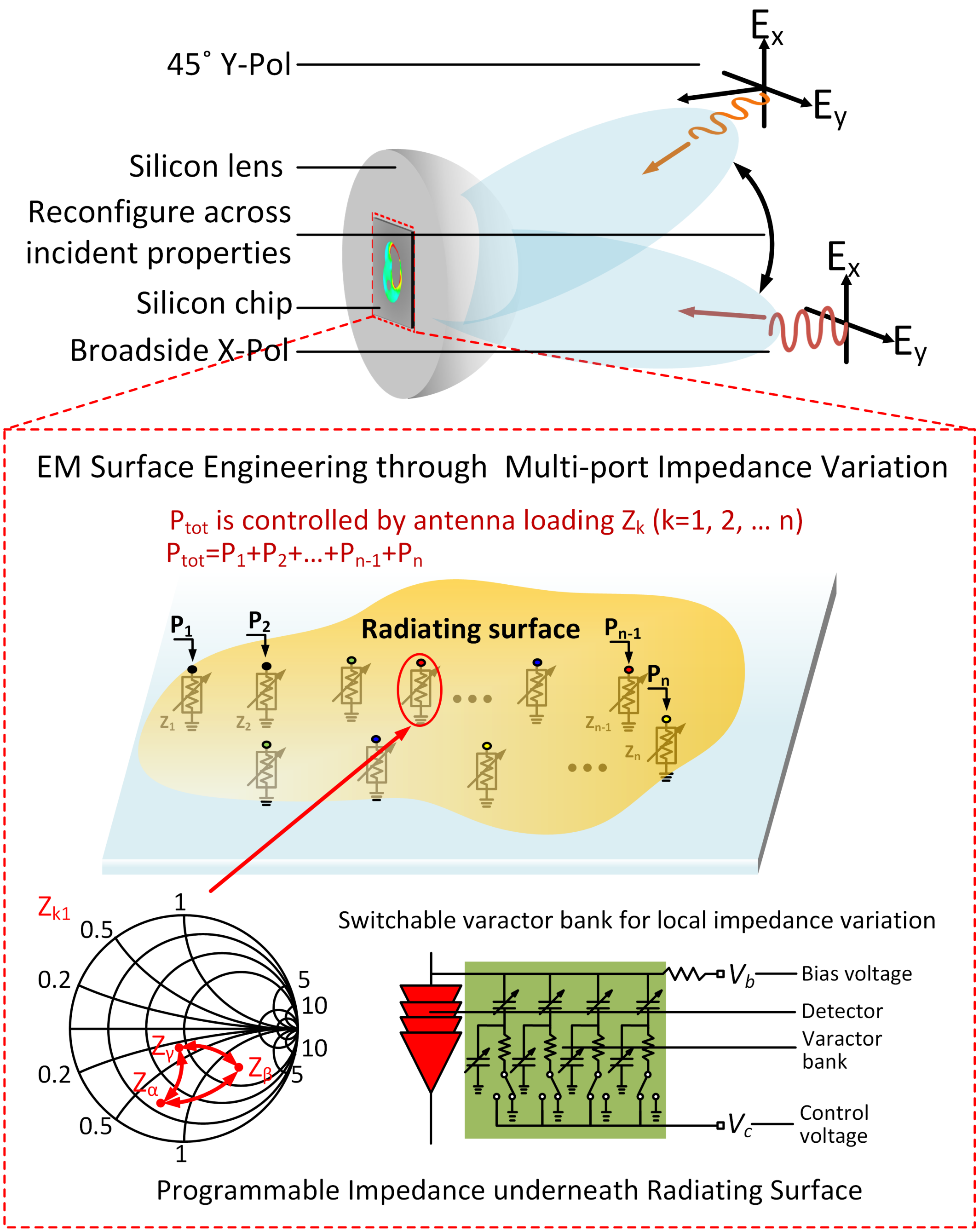

Figure 1: Reconfigurable THz sensor with subwavelength detection and reconfiguration across 0.10-1.0 THz.

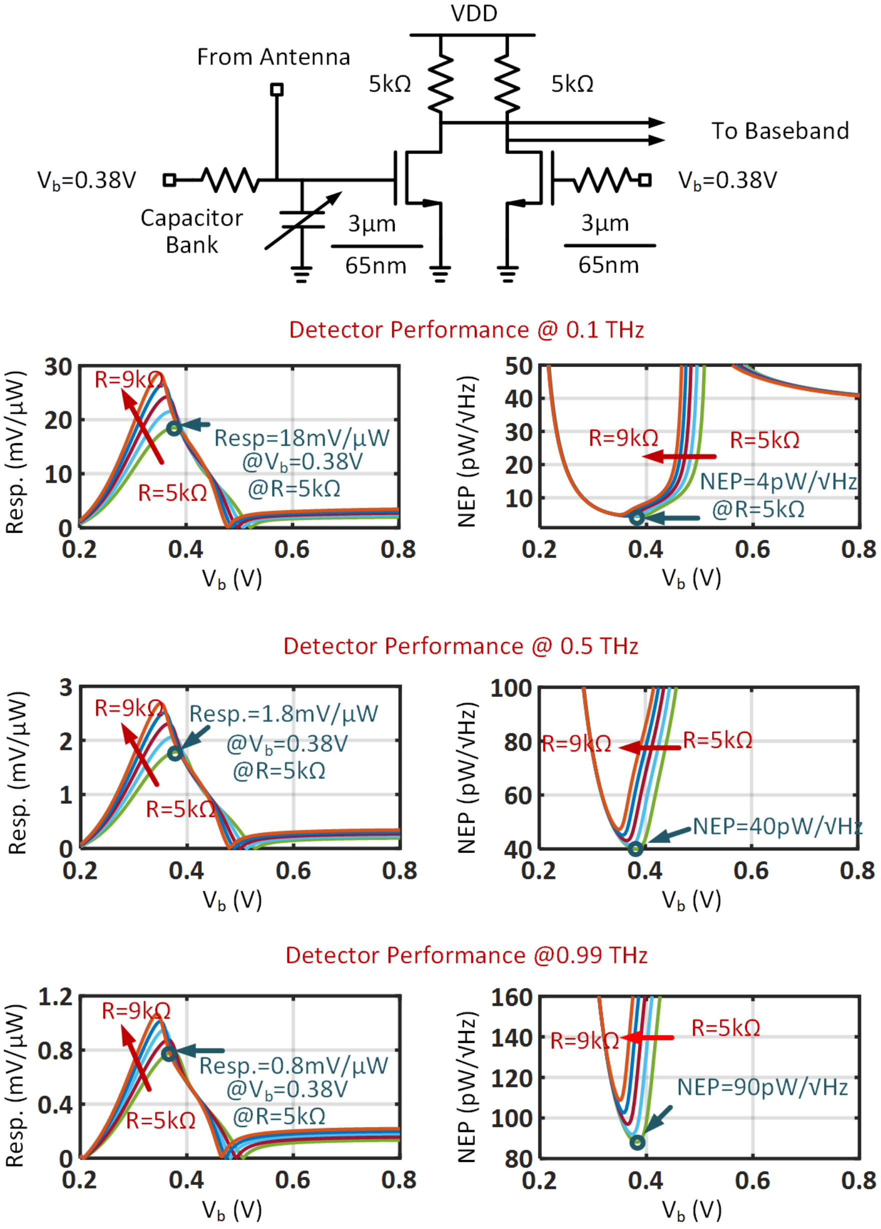

Figure 2: The variation of responsivity and NEP of the THz detector when the bias voltage (V_{b}) and resistors (R) are optimized across the frequency range (0.10-1.0 THz).

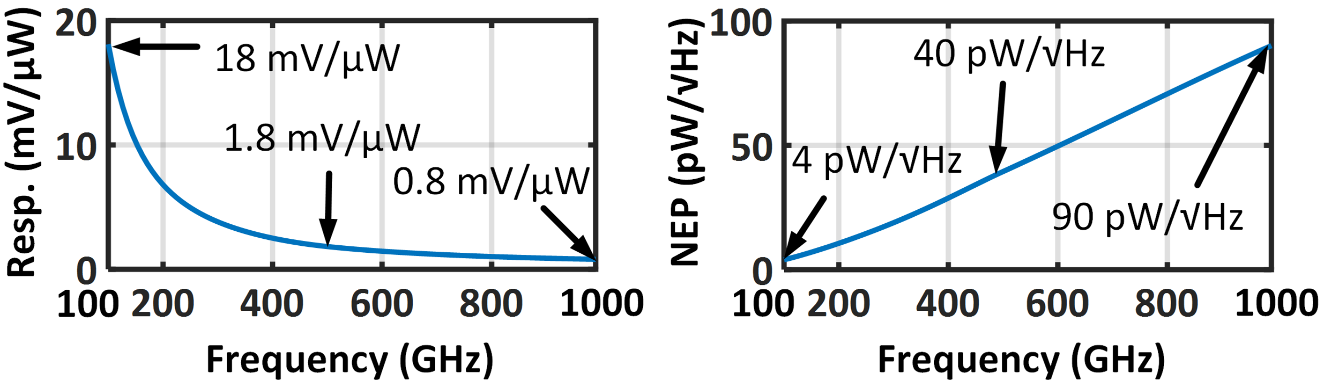

Figure 3: Optimized responsivity and NEP variation for a single detector.

varies from 4 pW/$\sqrt{Hz}$ to 90 pW/$\sqrt{Hz}$ over the frequency range.

The input impedance of the detector is programmable with a capacitor bank realized with multiple digitally controlled NFETs in parallel as shown in Fig. 1. The sizes of the NFETs are selected to be the same as the detector size to provide enough variation in the input impedance. The voltage V_c allows for optimization of the range of impedance variations across the frequency. In this design, we select V_c to be 1 V. The control signal of the capacitor bank is a 4-bit thermometer code provided by the 64-bit shift register. Supplementary Fig. 4 demonstrates the simulated variation of impedance across the frequency range when the switch settings are configured to the two extreme cases (1111: corresponding to all ON and 0000: corresponding to all OFF). The local impedance variations in the imaginary part of the impedance (see Supplementary Fig. 4) across the 16 detectors redistributes the surface current over the log-periodic tooth antenna for performance optimization.

To reduce $1/f$ noise during measurement, the incident field is chopped at a frequency higher

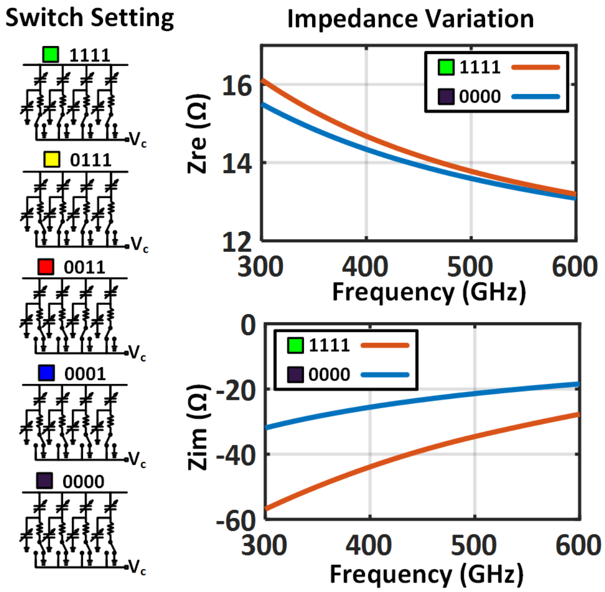

Figure 4: The simulated impedance change across the frequency range when the switch settings are configured to the two extreme cases (1111 and 0000).

than the $1/f$ noise corner of the detector. Supplementary Fig. 5 shows that simulated noise corner to be around 100 kHz. The chopped output signals from the detectors of the chip are then processed by the bandpass amplifiers on-chip and then by an external lock-in amplifier.

2 Supplementary Note 2: Baseband Amplifier

Supplementary Fig. 6 shows the circuit of the baseband amplifier. It consists of two stages of operational amplifiers connected with a feedback circuitry. In the operational amplifier, a complementary structure is used as the input pair to handle nearly rail-to-rail swings. The two stages of amplifiers provide a combined simulated gain of 80 dB with a lower cut-off frequency of 1 kHz and a higher cut-off frequency of 500 kHz as shown in Supplementary Fig. 7. The gain of the amplifier can be controlled by transistors biased in subthreshold region in a feedback cir-

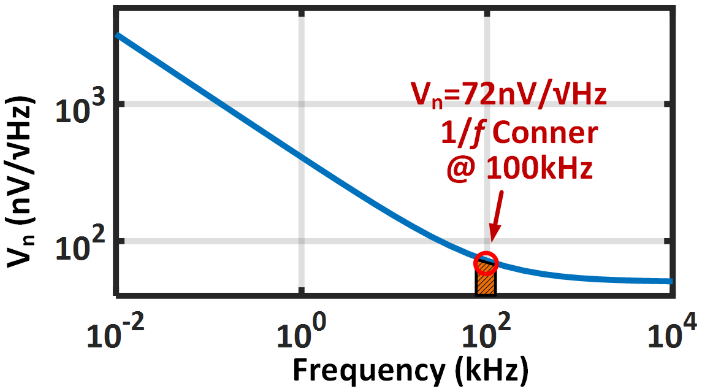

Figure 5: Simulated noise spectrum of the detector output showing that the $1/f$ noise corner to be around 100 KHz.

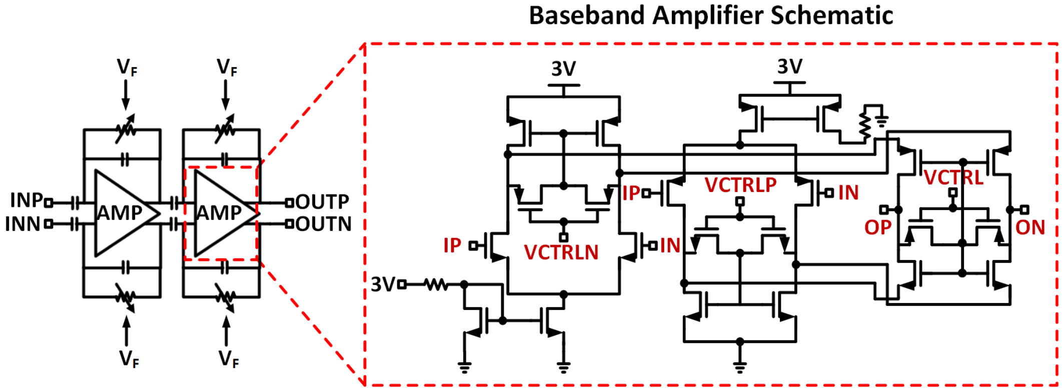

Figure 6: The schematic of the baseband amplifier which consists of two stages of operational amplifiers connected in a feedback circuitry. It has a tunable gain of 80 dB.

cuitry. Supplementary Fig. 7 also demonstrates a 46-80 dB tunable gain when the control voltage ($VCTRL$) is changed from 0 V to 1.5 V. This allows for enhancement in the dynamic range. The lower cut-off frequency of the baseband amplifier can also be controlled by another transistor biased in subthreshold as shown in the Supplementary Fig. 6 to vary between 1-20 KHz. This can be used for noise shaping of the output.

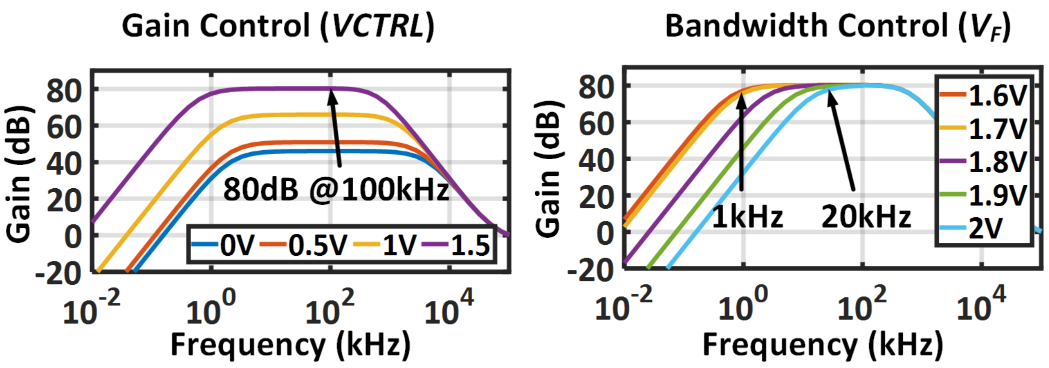

Figure 7: The simulated gain of the baseband amplifier and its tunable range.

3 Supplementary Note 3: Multi-port Antenna and Detector Power Calculation

Matching of a multi-port antenna to the multi-port detector array for calculating power absorption can be evaluated from Supplementary Fig. 8. The power absorption into the multi-port detector system can be evaluated from

$$P_{\text{rec}} = \frac{1}{2}\text{Re}\left(\mathbf{V_L^H I_L}\right) \tag{1}$$

$$\mathbf{V} = \mathbf{V_L} + \mathbf{Z_{Ant}I_L} = \mathbf{Z_L I_L} + \mathbf{Z_{Ant} I_L} \tag{2}$$

Using Supplementary Equations (1) into (2), we get

$$P_{\text{rec}} = \frac{1}{2}\text{Re}\left(\mathbf{V^H I_L} - \mathbf{I_L^H Z_{Ant}^H I_L}\right) \tag{3}$$

Replacing $\mathbf{I_L} = (\mathbf{Z_{Ant}} + \mathbf{Z_L})^{-\mathbf{1}}\mathbf{V}$ in (3) and simplifying, we obtain equation (1) in the manuscript.

The motivation of direct digital programming of the antenna surface with the multi-port approach goes to simultaneous reconfiguration of all the properties of the incident fields ie. frequency, angle of incidence and polarization, implemented with detectors that by themselves are not capable of large reconfiguration (hence the distributed approach). However, it is instructive to compare only the bandwidth with a single port log-periodic tooth antenna. This has to be done with a comparable matching network in mind.

In Supplementary Figure. 9, we do this against the classical log periodic tooth antenna coupled to a detector with 2nd and 4th order matching networks. The element quality factors of are taken to be 15 which are typically optimistic quality factors in the range of 100-1000 GHz. As can be seen in comparison with Fig. 5f (in the manuscript), that the reconfigurable range for the multiport structure exceeds that of the single port design. This is in addition to the ability to reconfigure the reception beam against incidence angles and polarization. in terms of frequency response is shown in Supplementary Fig. 9.

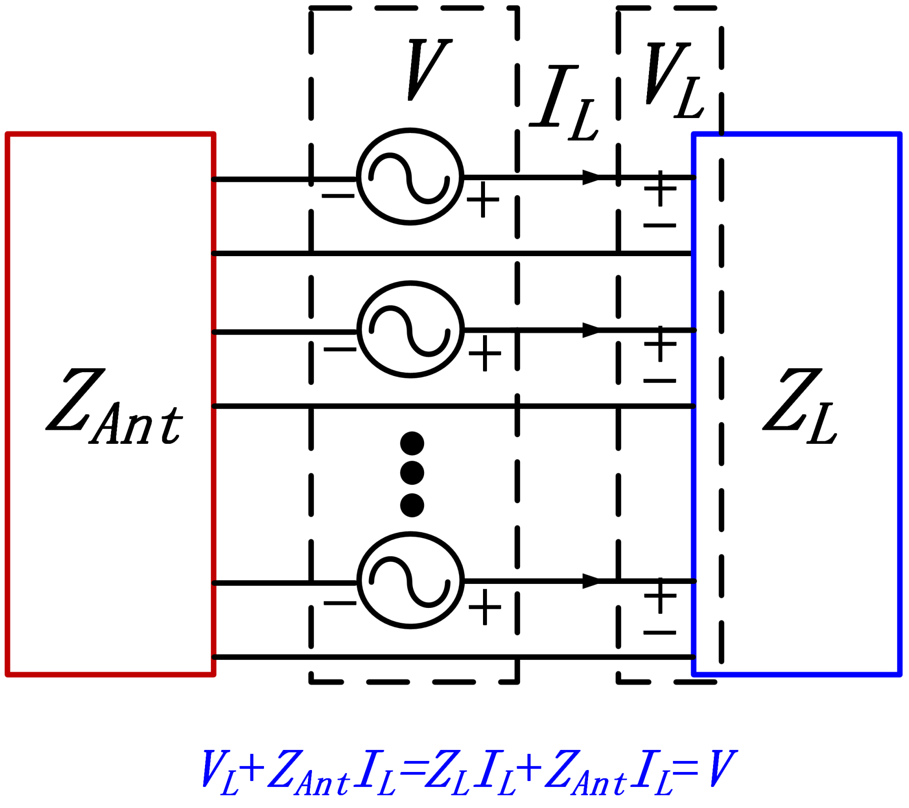

Figure 8: Multi-port power absorption.

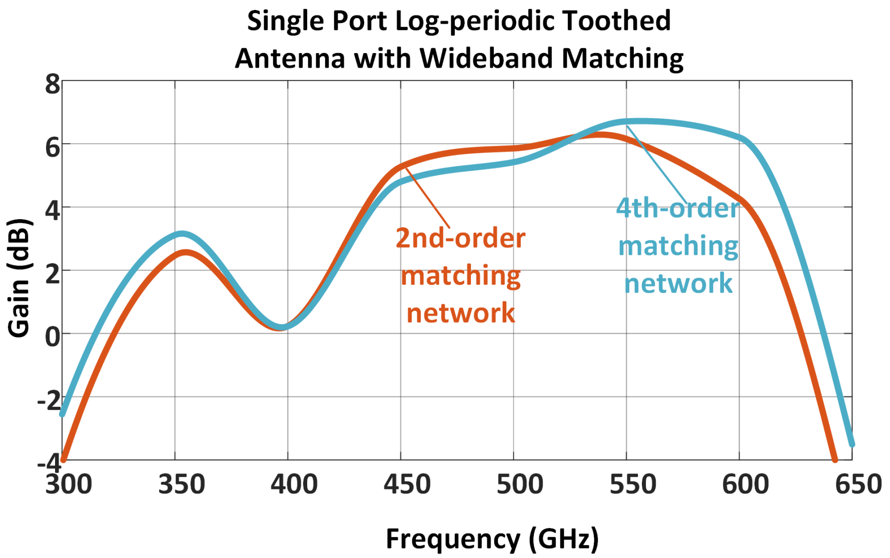

Figure 9: Frequency response of a single port log-periodic tooth antenna with second and fourth-order matching networks.

4 Supplementary Note 4: Measurement setup

The responsivity and NEP of the system across frequencies, incident angles and polarizations are characterized using the measurement setup shown in Supplementary Fig. 8. The input signals across 0.1-1.0 THz are generated with a chain of frequency multipliers driven by Agilent 8257D RF signal generator. The signal generator produces a RF signal below 40 GHz which is chopped at 100 kHz. A chain of amplifiers and frequency multiplier set is used to create the chopped signals between 0.1-1.0 THz. The signals are radiated by horn antennas to be incident on the chip from the side of the silicon lens. The outputs of the signals are processed by a lock-in amplifier which are read out through GPIB controls into a computer. Both the lock-in and the signal generator are synchronized to an external reference signal. The chip is set on a rotational stage to allow for variations of the angles of incidence. The gradient descent algorithm is calculated in the computer and instructions for reconfiguration are set via a FPGA into the chip. The process is fully automated that allows autonomous optimization of the chip setting for the given spectrum, polarization and angle of incidence of the radiated signal.

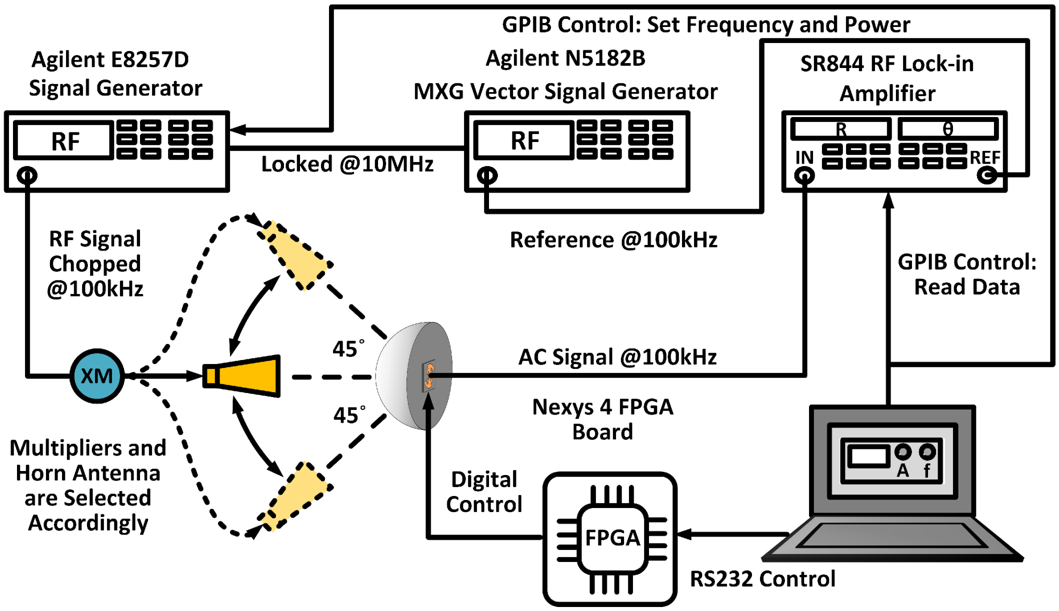

Figure 10: Measurement setup for chip testing across the three incident field properties: spectrum (0.1-1.0 THz), incident angles and polarization. The setup is fully automated to allow for autonomous sensor optimization for the given irradiation.
